# A retrospective case series on bisphosphonate related osteonecrosis of the jaw in 20 cats

**DOI:** 10.3389/fvets.2024.1436988

**Published:** 2024-08-23

**Authors:** Suzanna L. Hatunen, Jamie G. Anderson, Cynthia M. Bell, Hugo C. Campos, Matthew D. Finkelman, Bonnie H. Shope

**Affiliations:** ^1^Veterinary Dental Services LLC., Boxborough, MA, United States; ^2^School of Dental Medicine, University of Pennsylvania, Philadelphia, PA, United States; ^3^Specialty Oral Pathology for Animals, Geneseo, IL, United States; ^4^School of Dental Medicine, Tufts University, Boston, MA, United States

**Keywords:** bisphosphonate, alendronate, MRONJ, BRONJ, osteonecrosis, jaw, feline, hypercalcemia

## Abstract

**Introduction:**

This retrospective study highlights the salient aspects of a series of feline patients affected with bisphosphonate related osteonecrosis of the jaw. Though more commonly published in human literature, this presentation is rare in cats. The authors hope that this study will assist in making this a more globally known entity with subsequent improved prognosis.

**Methods:**

Data was retrospectively obtained from the medical records between 2015 and 2021 of 20 cats with Medication Related Osteonecrosis of the Jaw. Data included patient information, clinical history, presenting complaint, systemic diseases, details referable to hypercalcemia and treatment thereof, bisphosphonate specifics (dose and duration), clinical presentation of the lesion, diagnostic testing including radiographic and histopathologic descriptions, treatment, and outcome.

**Results:**

Pertinent results include that all 20 cats who developed Medication Related Osteonecrosis of the Jaw had been treated for idiopathic hypercalcemia with the bisphosphonate medication alendronate. Eighty-five percent of the cases had prior dental extractions at the site of MRONJ lesion. Ninety-five percent of the affected cats required a surgical procedure to control the disease. Thirty-five percent of cases required at least one revision surgery after the initial procedure was performed. Diagnosis of MRONJ was made by a correlation of diagnostic findings and patient history. No single diagnostic, or combination was pathognomonic for lesion diagnosis. As well, there were no statistically significant associations between patient variables assessed and the overall patient outcome.

**Discussion:**

The case series reveals that cats with feline idiopathic hypercalcemia treated with alendronate may be at a risk for development of MRONJ, a serious oral condition with significant morbidity. Prior dental extraction sites in patients concurrently treated with bisphosphonate medications were often associated with MRONJ lesions. Therefore, any needed dental surgery should be performed prior to the use of bisphosphonates where possible. The authors have also included a relevant comparative literature review.

## Introduction

Medication Related Osteonecrosis of the Jaw (MRONJ) (1), also referred to as Antiresorptive Agent Related Osteonecrosis of the Jaw (ARONJ) (2) and formerly known as Bisphosphonate Related Osteonecrosis of the Jaw (BRONJ) (1, 3), is a rare but intractable disease in humans linked with long term use of potent antiresorptive medications, such as bisphosphonates (BP) and denosumab (1, 4–6), and angiogenesis inhibitors (7). The American Association of Oral and Maxillofacial Surgeons (AAOMS) has preferred the term “medication related osteonecrosis of the jaw” (MRONJ) to include other antiresorptive and antiangiogenic drugs that have also resulted in necrosis of the mandible, maxilla or both (1). MRONJ secondary to BP use has been similarly described in the literature in a single cat (8), dogs (9–11), mice (12), rats (13–15), sheep (16) and mini-pigs (17, 18). BPs are a class of antiresorptive drug that have a high affinity for bone. They are small molecules integrated within the bone matrix and have a long half-life in the skeleton in humans and dogs (19). Accumulation of BPs within the bone matrix results in osteoclastic apoptosis and reduced bone resorption, which alters the normal physiological remodeling of the bone. Due to the reduced potential to remove senescent bone, there is an increased risk for necrotic bone accumulation (1, 6). The pathophysiology of MRONJ remains unknown. There are a few theories on MRONJ pathogenesis, though it is likely that the pathophysiology of the disease is multifactorial (1).

The AAOMS 2022 Position Paper defines MRONJ as: (1) current or previous treatment with antiresorptive therapy alone or in combination with immune modulators or antiangiogenic medications, (2) exposed bone or bone that can be probed through an intraoral or extraoral fistula(e) in the maxillofacial region that has persisted for more than 8 weeks and (3) no history of radiation therapy to the jaws or metastatic disease to the jaws (1). In humans, MRONJ is related to the cumulative dose of BPs, the length of treatment, the route of BP administration and the type of BP used (20), with a positive association for increased doses, longer duration of treatment, intravenous administration of BPs and nitrogen-containing BPs (1). In people, known risk factors for MRONJ include dental extractions, chemotherapy, periodontal disease, glucocorticoid therapy, denture and implant use, erythropoietin therapy, tobacco use, increasing age, sex (predilection for females), location in the maxillofacial region (predilection for the mandible) and systemic diseases such as diabetes, cancer, and hyperthyroidism (1, 5, 6, 21, 22). The literature reveals that dentoalveolar surgery is the most common predisposing factor to MRONJ, with tooth extractions being the inciting cause reported between 62–82% of cases (1). Further, the prevalence rates of MRONJ reported in humans varies between 0 to 0.35% (5). The risk of MRONJ is considerably higher in patients that are on BPs to treat underlying malignancies between 0.7 to 18% (1, 5, 23), whereas the incidence in patients with osteoporosis ranges from 0.001 to 0.05% (1, 5, 23). With appropriate treatment, high success rates for the treatment of MRONJ have been reported in people (1, 5).

Hypercalcemia in cats is defined as total Ca greater than 10.8 mg/dL, or ionized Ca greater than 5.6 mg/dL (>1.4 mmol/L) (24). Causes of hypercalcemia include acute or chronic kidney disease, malignancy associated, idiopathic hypercalcemia, primary hyperparathyroidism, hypoadrenocorticism, toxin ingestion (e.g., cholecalciferol, calcipotriene), granulomatous disease and osteolytic disease (24–26). Idiopathic hypercalcemia is considered one of the most common explanations of hypercalcemia in cats. In cats, BPs are predominantly prescribed to treat idiopathic or refractory hypercalcemia (8, 25, 27–30). However, it has also been described in the treatment of tooth resorption (29, 30). Anecdotally, they have also been used in the treatment of feline oral squamous cell carcinoma.

The exact mechanisms of MRONJ in cats are unknown, published risk factors are limited, and no clear treatment strategies are published. A veterinary-focused literature review on BRONJ (3) was published over a decade ago, yet to the author’s knowledge, there have been no published case series investigating MRONJ in cats. This emphasizes that despite the published review article highlighting the clinical features of MRONJ to be aware of in our veterinary patients, it has taken over a decade to identify, aggregate and consolidate this medical information. The purpose of this retrospective descriptive study was to present a narrative account of MRONJ in cats that includes presenting concerns, clinical findings, diagnostic testing, interventions, outcomes and prognosis. Conclusions from the study offer insights into this rare, but potentially devastating disease in cats.

## Materials and methods

### Case selection

Twenty feline patients with diagnosed MRONJ secondary to bisphosphonate therapy were included in this sample from 2015 and 2021. Inclusion criteria for cats were if they had been on antiresorptive (bisphosphonate) medications, and their clinical signs and diagnosis for MRONJ were consistent with the AAOMS 2022 Position Paper definition of MRONJ (1). This included visible exposed bone, or bone that could be probed through a chronic fistula of more than 8 weeks. Exclusion criteria included any patient that had known neoplasia with metastasis to the jaw, primary oral neoplasia or a history of head and neck radiation.

### Medical history

Medical records from 20 feline patients with BP associated MRONJ were evaluated, and the data extrapolated. Data assessment fell into the following categories.

Signalment data: Patient age at time of MRONJ diagnosis, breed (distributed into purebred or non-purebred), sex and desexing status.

Patient data: Body weight (kilograms) at time of MRONJ diagnosis, the underlying diagnosis for BP treatment and if the patient had any prior radiation treatment.

Concurrent systemic diseases: Diabetes mellitus, renal disease, cardiac disease, gastrointestinal disease, Feline immunodeficiency virus (FIV), Feline leukemia virus (FeLV), hyperthyroidism, any known skeletal fractures during or after BP treatment, and/or any known metastatic bone disease to the jaws.

Specifics of BP medication: The type of BP, length of time on BPs at the time of diagnosis, dose of BP medication (in milligrams), frequency of BP medication administered (per week), and the method of BP administration (oral, intravenous). Whether the BP was stopped (‘drug holiday’) was also documented (assessed as group 1: BPs stopped prior to surgery, group 2: any time after the first procedure was performed, and group 3: not stopped during treatment period).

MRONJ lesion description: The patient’s presenting sign(s), evidence of exposed bone/fistula or bone that could be probed under anesthesia through an intraoral or extraoral fistula, location of the MRONJ lesion/s (categorized into right maxilla, right mandible, left maxilla, left mandible), if the lesion was associated with a prior tooth extraction site, and the suspected inciting cause of MRONJ.

Imaging modalities and assessment: Descriptive analysis of radiographic findings (CBCT, dental radiography, conventional CT) was assessed by a human oral-maxillofacial radiologist (HC) and described in the results.

Histopathologic features: Descriptive analysis of histopathological findings associated with MRONJ, from available biopsy reports, was assessed by veterinary pathologist (CB) and described in the results.

Bacterial cultures: Descriptive analysis from available culture and sensitivity reports of which microorganisms were present were performed.

Treatment of MRONJ lesion: Specifics of surgical intervention included the number and type of surgeries performed. Surgery was defined as a procedure that exposed the underlying bone and lesion through creation of a mucoperiosteal flap. Subsequent procedures included surgical debridement, mandibulectomy or maxillectomy to resect affected tooth and bone, and tooth extractions. Whether the patient received any adjuvant treatment was also recorded.

Treatment outcome: The outcome was assessed as group 1: clinical resolution, group 2: euthanized, group 3: lost to follow up or group 4: ongoing cases. Clinical resolution was defined as no evidence of clinical signs nor MRONJ lesions in the oral cavity. The recheck time frame varied in each patient and was at the clinician’s discretion. In patients where clinical signs persisted, the outcome was determined after all revision surgeries.

### Statistical analysis

Frequency distributions (counts and percentages) were calculated. An available-case analysis was undertaken, i.e., patients that had missing data for some variables were included in analyses of other variables for which they had valid data. Associations between categorical variables were assessed with Fisher’s exact test. Differences in continuous outcomes between two groups were analyzed with the independent samples t-test or the Mann–Whitney U test, depending on the normality of the data. Normality was determined with the Shapiro–Wilk test. The Spearman rank correlation was used to examine correlations between continuous variables. The significance level was set at α = 0.05. SPSS 28 (IBM Corp., Armonk, NY, United States) was used in the analysis.

### Ethics statement

Standard veterinary private practice hospitals, as opposed to veterinary medical teaching hospitals, do not employ Institutional Animal Care and Use Committee (IACUC). As such, the study conformed to the American Animal Hospital Association (AAHA) Guidelines for Dental Care and ethics (31). For all the cats in this study, all surgical procedures described were performed under general anesthesia with appropriate regional anesthesia and post-operative analgesic administration and followed the AAHA Guidelines for Dental Care and ethics.

## Results

Known cases of MRONJ in 20 feline patients diagnosed between 2015 and 2021 were obtained from 11 veterinarians of which 19/20 (95%) of veterinarians were Board Certified Veterinary Dentists™ working at either tertiary care facilities or private practice, and 1/20 (5%) of the veterinarians was a referring DVM in primary practice.

### Signalment data

The mean ± SD patient body weight was 4.3 ± 1.6 kg. 14/20 (70%) were non-purebreed, and 6/20 (30%) were purebreed cats. 9/20 (45%) cats were female, and 11/20 (55%) cats were male. 20/20 (100%) of cats were desexed.

### Patient data

The mean ± SD age of the patient at time of MRONJ diagnosis was 10.3 ± 3.8 years old. None of the patients had any history of prior radiation therapy or any known metastatic bone disease to the jaw, allowing all lesions to qualify as MRONJ.

### Concurrent systemic diseases

Out of the 19 cases that had data provided regarding concurrent diseases, 16/19 (84.2%) had known concurrent diseases, and 3/19 (15.8%) did not. Concurrent diseases investigated included diabetes mellitus (1/19, 5.3%), chronic renal disease (12/19, 63.2%), cardiac disease (9/19, 47.4%), hyperthyroidism (3/19, 15.8%), undetermined chronic gastrointestinal disease (6/19, 31.6%), FIV (2/19, 10.5%), and FeLV (0/19, 0%). Skeletal fractures during or after BP treatment was seen in 2/19, 10.5%.

### Specifics of BP medication

20/20 (100%) of the cases were on bisphosphonate medications to treat idiopathic hypercalcemia. 19/20 (95%) were on alendronate alone, and 1/20 (5%) was on alendronate at the time of diagnosis and had also been treated with a different bisphosphonate medication (pamidronate) prior. The results for 18/20 cases were provided for the total dose of alendronate (in milligrams) per week at the time of diagnosis. The mean ± SD weekly dose of alendronate per cat at time of diagnosis was 20.1 ± 12.3 mg. 17/18 (94.4%) of cases were on oral administration of alendronate and 1/18 (5.6%) was on oral administration of alendronate, with a prior history of intravenous pamidronate. The total time on bisphosphonates, provided in 15/20 cases, ranged from 5 months to 66 months, with the mean ± SD time being 30.2 ± 18.5 months. Sixteen cases provided data regarding whether bisphosphonate medication was stopped, which was analyzed in association with when treatment for MRONJ lesion started. Results are summarized in Table 1.

**Table 1:** Specifics of BP medication for each case.

Case (Patient)	Reason for BP Tx	Type of BP	Time of BP Medication (in months) at the time of initial MRONJ Clinical Signs	Dose of Alendronate (in milligrams) at the time of diagnosis	Frequency of Alendronate dose given (per week)	Method of administering BP medications	Whether Alendronate was stopped (‘drug holiday’) and when.
1	Idiopathic hypercalcemia	Alendronate	37	10	Once weekly	PO	Stopped during treatment period (after the first surgery)
2	Idiopathic hypercalcemia	Alendronate	20	20	Once weekly	PO	Stopped prior to initial sx
3	Idiopathic hypercalcemia	Alendronate	12	10	Once weekly	PO	Stopped prior to initial sx
4	Idiopathic hypercalcemia	Alendronate	11	10	Once weekly	PO	Stopped prior to initial sx
5	Idiopathic hypercalcemia	Alendronate	38	20	Once weekly	PO	Stopped during treatment period (after the first surgery)
6	Idiopathic hypercalcemia	Alendronate	54	20	Once weekly	PO	Was not stopped
7	Idiopathic hypercalcemia	Alendronate, prior history of Pamidronate	51	10	Three times weekly	PO, prior IV BP (Pamidronate)	Stopped during treatment period (after the first surgery)
8	Idiopathic hypercalcemia	Alendronate	9	20	Once weekly	PO	Stopped prior to initial sx
9	Idiopathic hypercalcemia	Alendronate	No data	10	Twice weekly	PO	Stopped during treatment period (after the first surgery)
10	Idiopathic hypercalcemia	Alendronate	No data	No data	No data	No data	No data
11	Idiopathic hypercalcemia	Alendronate	36	10	Once weekly	PO	Was not stopped
12	Idiopathic hypercalcemia	Alendronate	12	20	Once weekly	PO	Stopped prior to initial sx
13	Idiopathic hypercalcemia	Alendronate	No data	15	Once weekly	PO	Stopped during treatment period (after the first surgery)
14	Idiopathic hypercalcemia	Alendronate	66	10	Once weekly	PO	Stopped during treatment period (after the first surgery)
15	Idiopathic hypercalcemia	Alendronate	5	60	Once weekly	PO	No data
16	Idiopathic hypercalcemia	Alendronate	48	30	Once weekly	PO	No data
17	Idiopathic hypercalcemia	Alendronate	36	30	Once weekly	PO	N/A-Patient did not undergo surgery, euthanized.
18	Idiopathic hypercalcemia	Alendronate	No data	7.5	Once weekly	PO	Stopped prior to initial sx
19	Idiopathic hypercalcemia	Alendronate	25	20	Once weekly	PO	Stopped prior to initial sx
20	Idiopathic hypercalcemia	Alendronate	No data	No data	No data	No data	Stopped prior to initial sx

### MRONJ lesion description

The main presenting clinical signs included: oral discomfort, inappetence, facial swelling, chronic draining tract, drooling, abscess, bone exposure or a nonhealing site associated with prior extraction site. In 100% of cases there was evidence of exposed bone, or bone that could be probed through an intraoral or extraoral fistula(e). The dental quadrants affected by the MRONJ lesion were analyzed for each patient. The most common presentation was a focal lesion in one quadrant of the oral cavity. 16/20 (80%) had a focal lesion in only one dental quadrant. The distribution of lesions in the mandible or maxilla alone was the same (45%). 2/20 (10%) had lesions in both the maxilla and mandible. 17/20 (85%) had prior dental extractions at the same location as the lesion, and only 3/20 (15%) had teeth present in the site of the active lesion. These 3 cases included presence of tooth resorption and periodontal disease, tooth resorption and traumatic malocclusion, and periodontal disease alone.

### Imaging modalities and assessment

**Figure 1:**
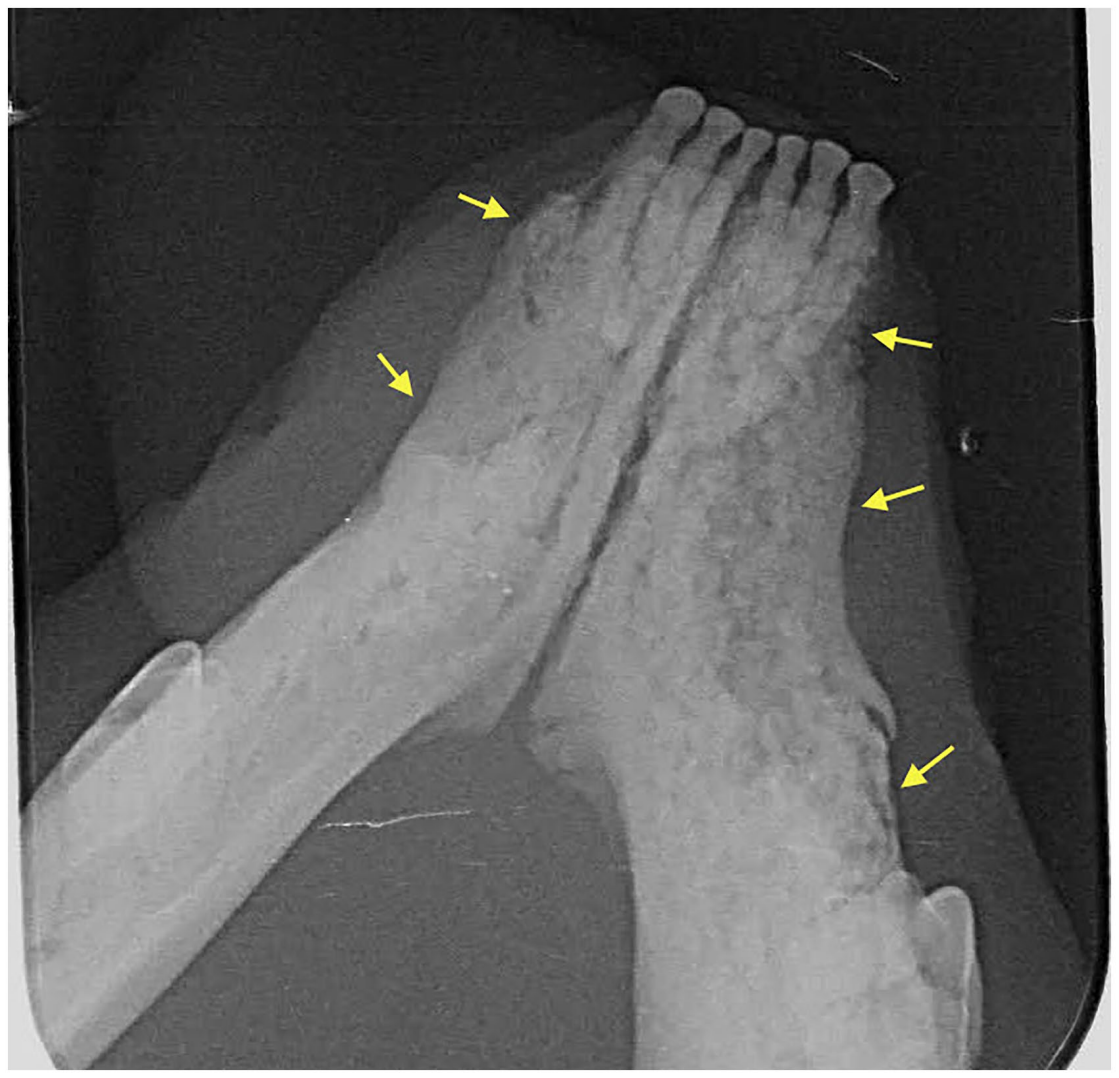
Case 7: Dental radiograph showing ill-defined, irregular shaped, low-dense radiopaque areas located at the most superficial aspect of the affected bone with irregular alveolar bone margins (yellow arrows).

**Figure 2:**
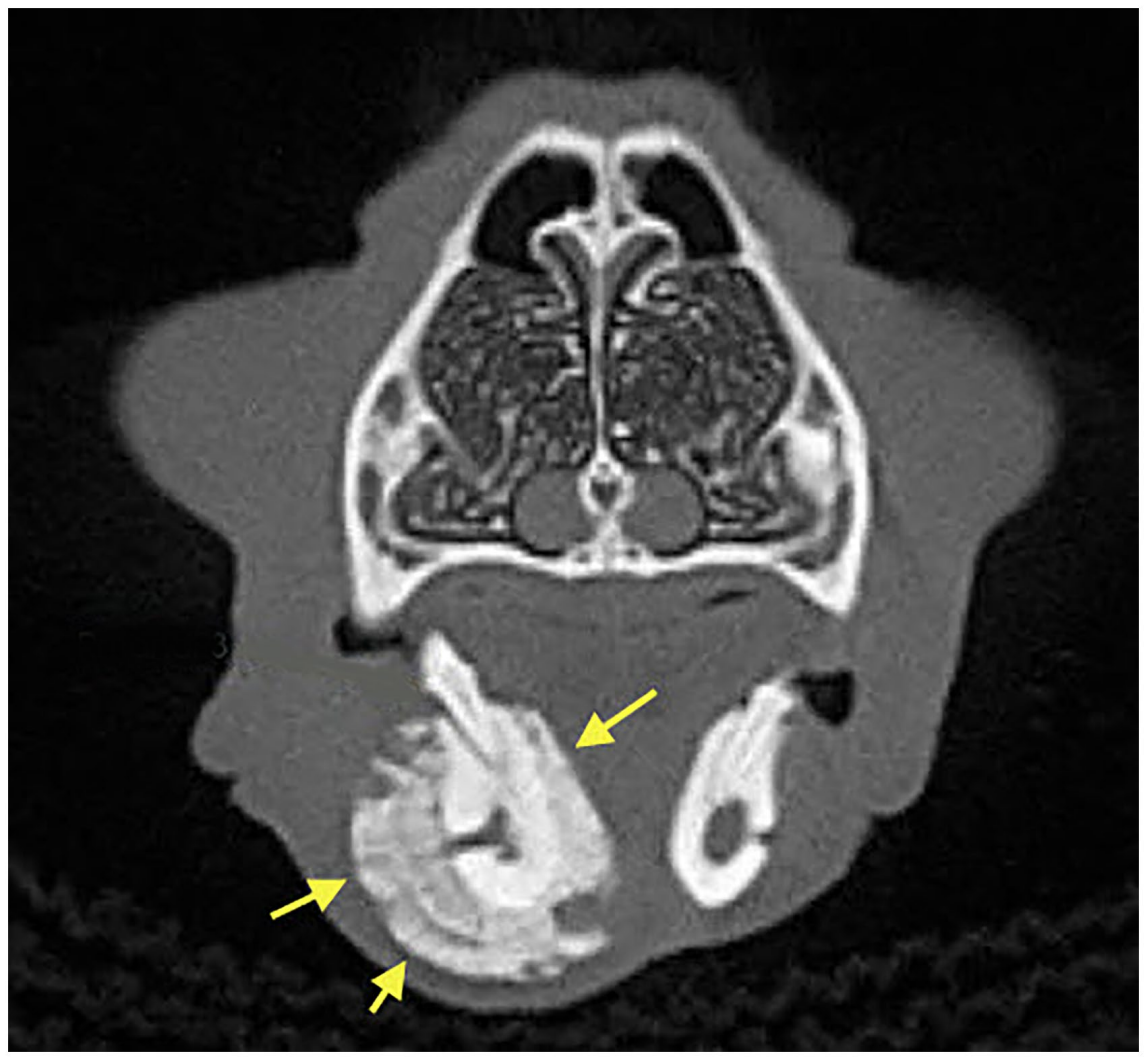
Case 8: 3D Rendered image of a CBCT scan showing solid periosteal reaction of the affected area (yellow arrows), almost double the size of the contralateral, non-affected region.

**Figure 3:**
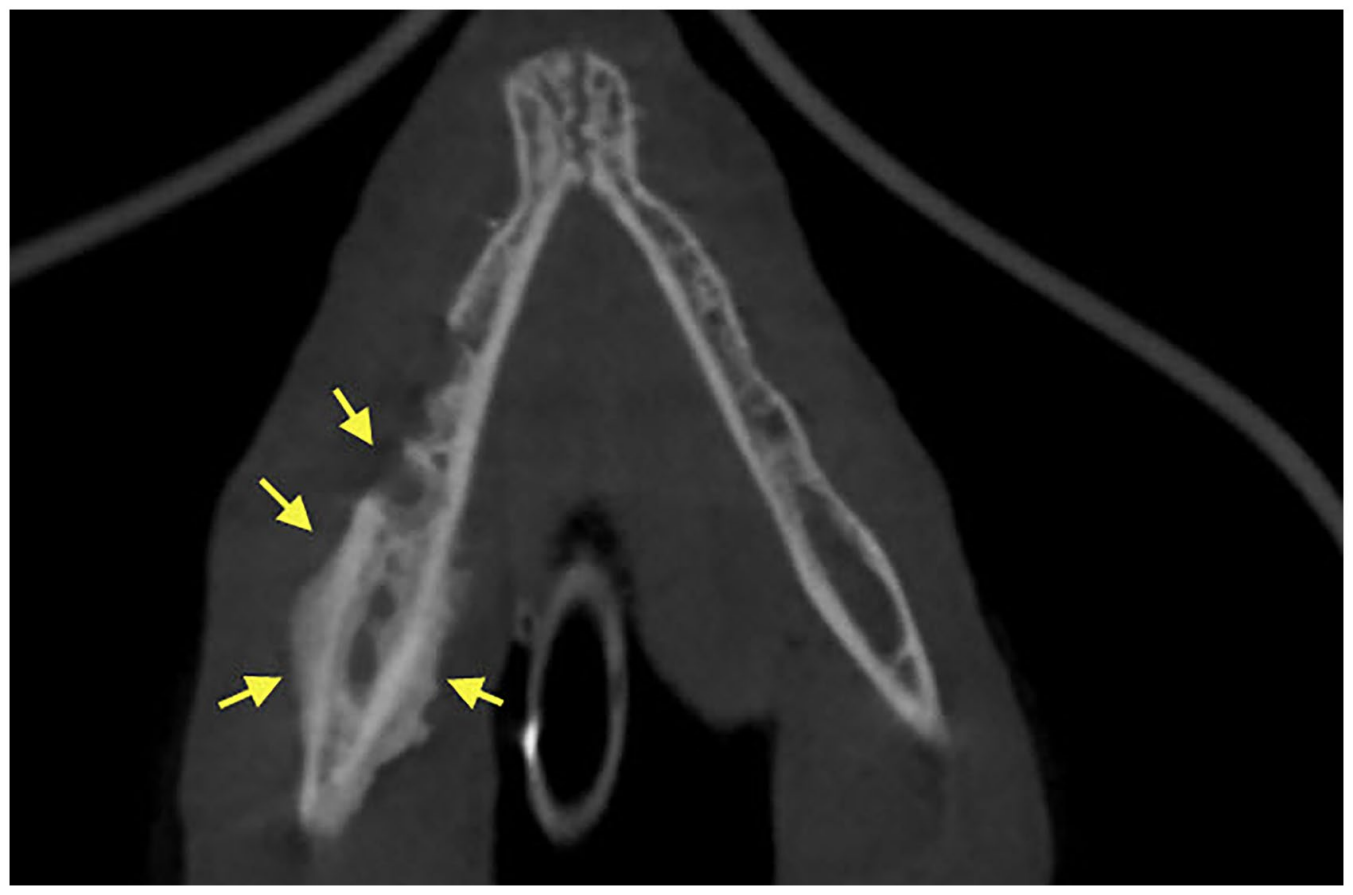
Case 1: 3D Rendered image of a CBCT scan showing non-healed dental alveolus with low-dense radiopaque areas in the alveolus and adjacent areas of solid periosteal reaction (yellow arrows).

Dental radiographs were performed in all 20 patients (100%). Cone Beam CT (CBCT) scan was performed in 6/20 (30%) of patients. Conventional CT was performed in 2/20 (10%) of cases. Radiographic evaluation was performed by a human Oral and Maxillofacial Radiologist on images provided. Prior to the radiographic assessment, a calibration session was performed to understand the normal radiographic appearance of feline teeth and anatomical structures. The radiographic evaluation was performed in a room with dimmed light, using high resolution displays (DELLU2413 Color Profile, D6500). CBCT were reviewed with the software Anatomage InVivo 6.5 (Anatomage, Santa Clara, CA). Multiplanar reformatted projections (transverse, coronal and sagittal views); sections of thickness 0.0 mm and interval of 0.1 mm were used to evaluate CBCT scans to avoid any superposition. The most common radiographic appearance noted in the 2D radiographic images and CBCT scans was an ill-defined, irregular shaped low-dense radiopaque area located at the most superficial aspect of the affected bone (Figure 1). Some specimens presented with enlargement of the affected area almost double the size of the non-affected region (Figure 2). One of the specimens showed non-healed dental alveolus with low-dense radiopaque areas in the alveolus and adjacent areas (Figure 3). Five cases presented with sequestra, consisting of non-vital bone surrounded by a radiolucent area. Radiographic appearance of MRONJ resembles the radiographic appearance of other entities as chronic osteomyelitis, osteoradionecrosis, and malignant neoplasm.

### Histopathologic features

**Figure 4:**
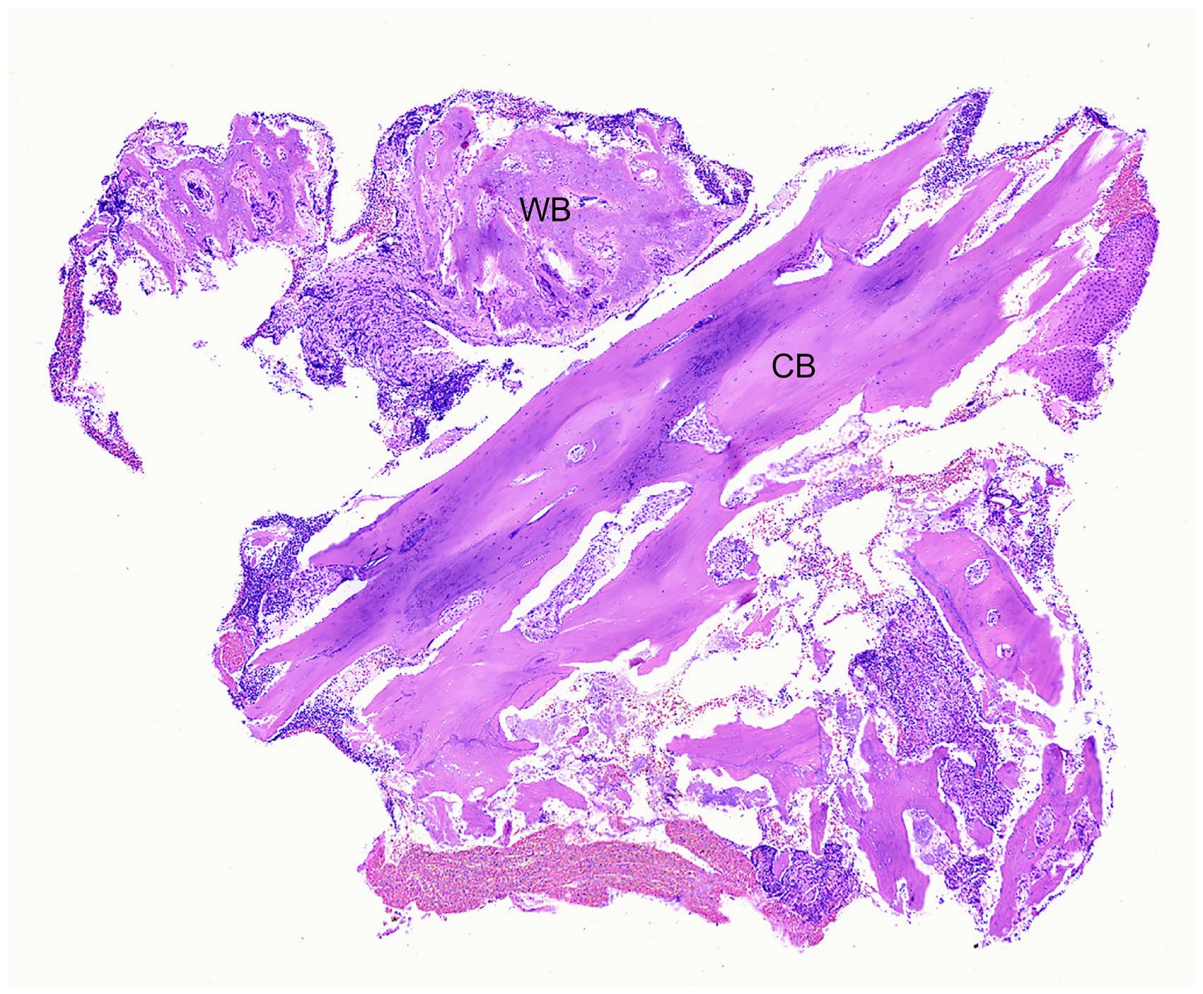
Case 1: Low magnification photomicrograph of bone removed from the right mandible, including cortical bone (CB) and periosteal new woven bone (WB). H&E stain.

**Figure 5:**
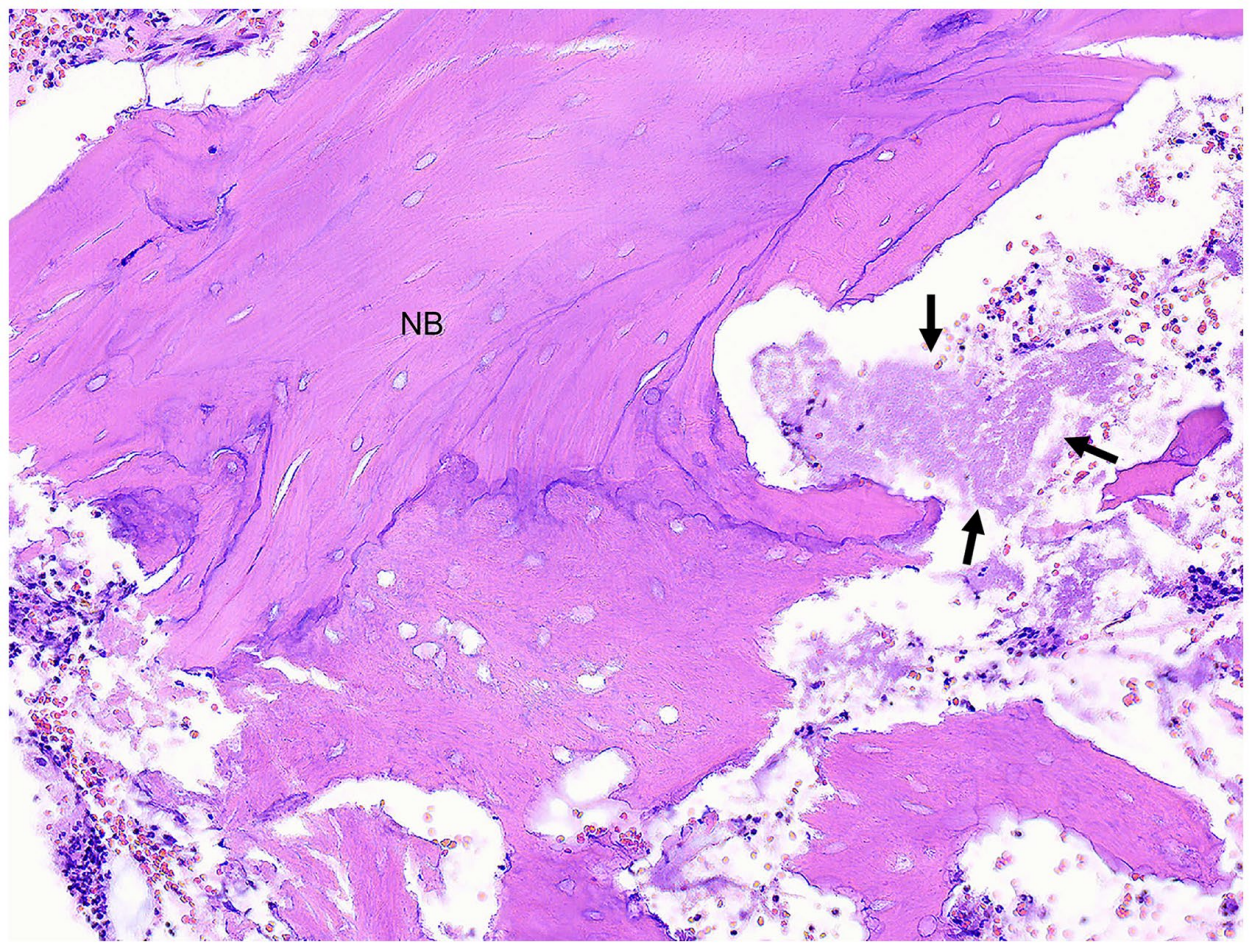
Case 1: Higher magnification photomicrograph of right mandibular bone shown in Figure 4. Some of the cortex is composed of necrotic bone (NB) in which osteocytes are absent and the edges of bone are irregular due to lysis. The fibrovascular stroma has been replaced by necrotic debris with clumps of bacteria (arrows) and degenerate neutrophils. H&E stain.

Histopathology of the lesion was performed in 17/20 (85%) patients. Analysis of data was based on firsthand microscopic evaluation of the biopsy specimen in 7/17 (41%) patients or from the histopathological description and diagnosis provided by another pathologist in 10/17 (59%) patients. Chronic osteomyelitis was consistent among biopsy samples from all patients. Osteomyelitis was commonly described as neutrophilic 12/17 (70%) and less often as pyogranulomatous 2/17 (12%), both neutrophilic and pyogranulomatous 2/17 (12%), or there was no mention of the type of inflammation 1/17 (6%). Necrotic bone and bacteria were identified in biopsy samples from 12/17 (71%) patients and 13/17 (76%) patients, respectively. Abundant mixed bacterial colonies were commonly seen in association with necrotic bone. The organization of bone reflected chronic, lytic, and proliferative remodeling (Figures 4, 5). Mucosa and gingiva that were overlying or adjacent to the affected bone were variably ulcerated. Neoplastic tissue was not seen in any of the samples.

### Bacterial cultures

18/20 (90%) patients provided data on whether bacterial culture and sensitivity testing was performed, and 8/18 (44%) patients had culture and sensitivity testing done on the lesion. The results showed aerobic isolates were present in all eight samples, which included *Pasturella* spp., *Burkholderia cepacian*, *Staphylococcus felis*, *Neisseria* spp., *Escherichia coli.*, *Actinomyces* spp., *Bacillus* spp., *Peptostreptococcus* spp., *Bacteroides* spp., *Corynebacterium* spp. and *Micrococcus* spp. Anaerobic isolates were identified in 4/8 (50%) of the cases, identifying *Bacteroides* spp., *Actinomyces* spp., *Porphyromonas* spp., *Propionibacterium* spp. and *Prevotella* spp. *Actinomyces* spp. was identified in 3/8 (38%) cases in our study. Importantly, no multidrug resistant isolates were identified.

### Treatment of MRONJ lesions

Aggressive surgical debridement of the affected bone and soft tissue, and any necessary extractions of teeth in the affected region were performed in 19/20 (95%) patients. One cat needed a follow up right mandibulectomy due to refractory MRONJ. 7/20 (35%) needed at least one revision surgery after the initial procedure was performed. One patient received no treatment. 12/20 (60%) underwent 1 surgery, 4/20 (20%) needed 2 surgeries, 1/20 (5%) needed 3 surgeries, 1/20 (5%) needed 5 surgeries and 1/20 (5%) needed 9 surgeries performed. Only one case provided information for any adjuvant therapy being performed. This patient received cold laser treatment, followed by hyperbaric oxygen treatment. Most patients (85%) also received antibiotics postoperatively.

### MRONJ treatment outcomes

Full clinical resolution (as described in the methods section) was achieved with surgery in 9/20 (45%) of cases. 2/20 (10%) were euthanized. However, it is unknown if the patients were euthanized due to the MRONJ lesions or other causes. 2/20 (10%) showed persistent clinical signs associated with MRONJ after surgical treatment and were ongoing cases at the time of writing, medically managed with long-term antibiotics. 7/20 (35%) patients were lost to follow up. 3/7 (43%) patients who were lost to follow up showed signs of reoccurring clinical signs and physical exam findings consistent with the original MRONJ lesion at recheck between 3 and 6 months postoperatively and were placed on extended courses of oral clindamycin. No further follow up for these patients was noted, so clinical outcome is unknown. Notably, the patients that had the most surgeries (3, 5 and 9 procedures respectively) and the patient who had a right mandibulectomy performed all had positive clinical outcomes.

### Prognostic outcomes

There was no statistically significant association with patient age (*p* = 0.38), gender (*p* = 0.45), body weight (*p* = 0.40), cat breed category (*p* = 0.49), concurrent underlying diseases (*p* = 0.18), such as diabetes mellitus (*p* = 1.0), chronic renal disease (*p* = 0.11), cardiac disease (*p* = 1.0), hyperthyroidism (*p* = 1.0), chronic gastrointestinal disease (*p* = 0.18), FIV and FeLV (*p* = 0.18) with patient outcome. There was also no statistically significant association between alendronate versus alendronate plus prior pamidronate (*p* = 1.0), oral versus intravenous administration of the bisphosphonate medication (*p* = 1.0), the weekly dose (in milligrams) of alendronate (*p* = 0.17), the frequency of alendronate administration weekly (*p* = 1.0), the length of bisphosphonate treatment at the time of lesion diagnosis (*p* = 0.09), and when alendronate was stopped in respect to if/when the patient had surgery (*p* = 0.50) with the patient outcome. There was also no significant association between the weekly dose (in milligrams) of alendronate (at the time of diagnosis) and the number of surgeries performed on a MRONJ lesion (*p* = 0.61). There was no statistically significant association between the outcome with which dental quadrant had MRONJ (right maxilla *p* = 0.49, right mandible *p* = 0.49, left maxilla *p* = 1.0, left mandible *p* = 0.18), if there was more than one dental quadrant affected (*p* = 1.0), the suspected inciting cause of the MRONJ lesion (*p* = 1.0), use of adjuvant treatment (*p* = 1.0), use of postoperative antibiotics (*p* = 1.0), and of the patients who had surgical treatment, the number of surgeries (*p* = 0.15) or the type of surgery (*p* = 1.0) performed.

## Discussion

Bisphosphonate use in people is well documented in the literature (1, 5, 32), and its clinical use in cats (3, 8, 27), dogs (10, 11, 33) and horses (34, 35) has also been recognized. The overall incidence of MRONJ after extractions in humans is relatively low (1), and the incidence of MRONJ in a series of cats has not yet been investigated. We determined that MRONJ is a potentially devastating complication of long-term BP treatment in cats. These findings will inform general veterinarians, internists and oncologists about the dangers of prescribing bisphosphonates in cats.

BP associated side effects are common in people and may be important in felines. In people, side effects other than MRONJ include erosive esophagitis, esophageal stenosis, uveitis, gastric ulcers, abdominal pain, and recurrent oral ulcers and blisters (36). Atypical femur fractures have also been described in people associated with longer duration of BP use (37). As well, fractures occurring during BP treatment were shown to be associated with a lower patient bone mineral strength index (BMSI) (38). Comparatively for cats, alendronate treatment at 5-20 mg/cat once weekly was well tolerated (25, 28). Most cats displayed no major side effects associated with chronic use. A single study documented severe hypophosphatemia with chronic alendronate, prompting treatment discontinuation (28). The authors recommend that frequent serum phosphate level assessment is appropriate. Of interest and importance, two of the case study cats had skeletal fractures. One had bilateral tibial fractures secondary to suspected osteopetrosis. The other case had both tibia and fibular fractures. In both cases, the limb fractures occurred after long-term BP treatment. Other studies have described insufficiency fractures in cats secondary to chronic alendronate use, with one study showing bilateral patellar fractures associated with osteosclerosis of both patellae (39), and another study describing a right calcaneal fracture and a left tibial fracture (29). In both cases alendronate was discontinued. Interestingly, these bones have also been described to fracture in the disease process Patellar Fracture and Dental Anomaly Syndrome (PADS) in cats; a connection between skeletal fractures in PADS cats, and cats with skeletal impact secondary to MRONJ is unknown and may warrant further investigation (40).

Anatomical and physiological features of the jawbones are likely a contributing factor to their predisposition for MRONJ in people and felines. In general, turnover of alveolar bone in both the mandible and maxilla are much greater than in the long bones, and are constantly exposed to chronic microtrauma from the forces of mastication (6). The mandibular and maxillary bones are separated from the oral cavity by thin mucosa, and trauma to this soft tissue can cause exposure of the bone to the external environment including microbes within dental plaque (4). Rat studies support the triggering role of infection in the onset of MRONJ (14, 15, 41). In humans, MRONJ occurs more often in the mandible than in the maxilla, though it can occur in both concurrently (1). Interestingly, in this current study of cats we found that it was more common for either the maxilla or mandible to be affected rather than both, but contrary to people, both the maxilla and mandible were equally affected when the lesion only affected one jaw (45%). In light of these findings, the authors emphasize the importance of the clinician performing a methodical examination of both jaws during routine periodontal treatment for cats on BP medications.

Referrable to risk factors for MRONJ in various systemic diseases, recent systematic reviews describe that the most common patient risk factors in people include concurrent chemotherapy, glucocorticoids, diabetes mellitus, hypertension, smoking and thrombin coagulopathies (6, 42). Our study shows that most of the cats had other concurrent systemic disease(s) present. With the current number of cat cases and without a control group, it is difficult to derive any clinical conclusion from our study between underlying diseases and how they may affect MRONJ development or outcome. It is well documented in humans that cancer patients receiving strong antiresorptive medications are at high risk to develop MRONJ (1, 43). Aging has been shown to be a significant risk for developing MRONJ in humans (5, 44). We could not determine if age was a risk factor in our case series. However, a future prospective study investigating the risk factors and prevalence of MRONJ lesions in all feline patients prescribed alendronate may find these factors influential.

**Figure 6:**
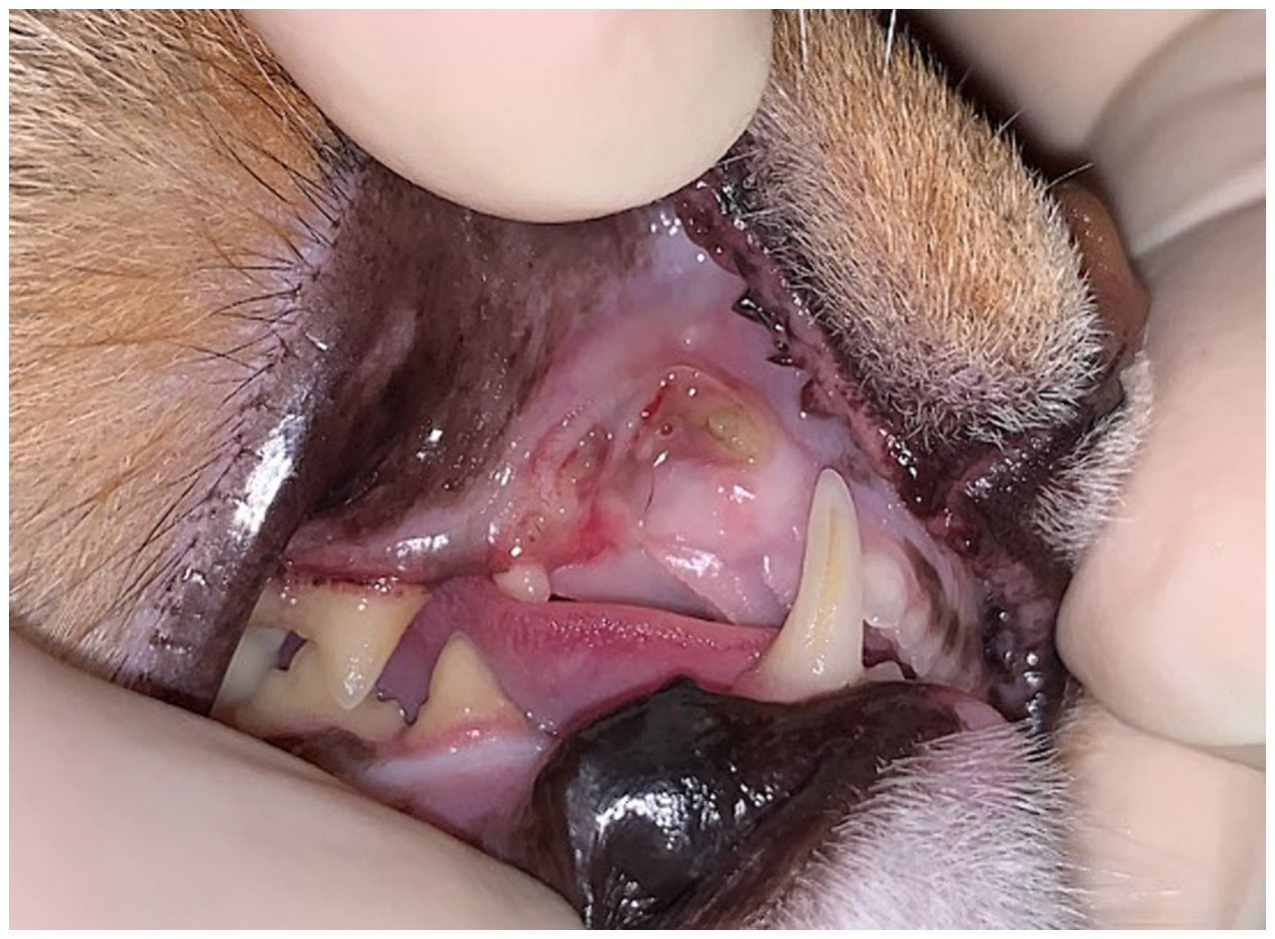
Case 2: Focal MRONJ lesion in a cat associated with prior extraction site (right maxillary canine tooth), with a region of chronic, infected non-healing lesion and a fistula which probes to underlying bone.

Patient history and clinical examination remain the most sensitive diagnostic means for MRONJ (5). Clinical appearance and symptoms/signs of MRONJ in humans and cats differ. Clinically in people the spectrum encompasses necrotic bone exposure ranging in a few millimeters to larger exposed areas, nonhealing extraction sites, soft tissue swelling, abscesses with fistulas and diffuse pain (5, 6). Paraesthesia of the lip has been described as an early sign of MRONJ (4). Lesions may remain asymptomatic for weeks to years, until a triggering event occurs (6, 45). In our patient population, the main presenting clinical signs for MRONJ included oral discomfort, inappetence, facial swelling, chronic draining tract, drooling and swelling, abscess, bone exposure or nonhealing associated with a prior extraction site. The most common presentation was a focal lesion in one quadrant of the oral cavity, associated with a prior extraction site (Figure 6). While the authors feel as though the clinical signs were most likely associated with underlying MRONJ lesion, it is possible that concurrent systemic diseases were also contributing to these clinical signs, particularly in less lesion-specific clinical signs such as inappetence. Unfortunately, in animals, the more subtle clinical signs such as paraesthesia of the lip may be difficult to assess and may lead to delayed diagnosis of this disease.

Other diagnostic modalities differ in their scope between people and felines. Imaging (dental radiographs, CBCT, conventional CT scan), histopathology and culture and sensitivity are significant in ruling out other causes of osteonecrosis of the jaw in either species. Focusing on radiography, findings in our study were highly variable in cats MRONJ lesions. They differed from ill-defined to irregular shaped radiopaque lesions, to significant alveolar bone enlargement of the affected region. Comparatively, cats with advanced stages of periodontitis may manifest as alveolar osteomyelitis and radiographically appear as a distinct mass effect and/or expansion of alveolar bone (46). Therefore, underlying periodontitis and infection likely contribute to the severity of radiographic bone expansion and profound osteoproliferation in some MRONJ lesions. It is important for the reader to understand these varied radiographic presentations, and to know that these findings are not pathognomonic to this disease, thus patient history and other diagnostics must be used to rule out other differentials. When MRONJ is suspected, radiographic evaluation with advanced imaging techniques such as CBCT or conventional CT is recommended to evaluate the affected areas without superposition. Given that conventional CT is superior for soft tissue imaging with the capacity to perform contrast studies (47), this modality may be advantageous over CBCT in diagnosing these jawbone lesions.

The histological findings in our study are consistent with the human literature (4, 5, 36). Neutrophilic osteomyelitis is characteristic of bacterial infection, although presence of bacteria in these lesions is presumed to be secondary to necrosis. The absence of necrotic bone in biopsy samples from some patients was likely the result of tissue sampling. Clinicians may have preferentially sampled vital bone, to better characterize the type of inflammation and rule out neoplasia. It is unlikely that the lack of necrotic bone in the incisional biopsy samples indicated true absence of jaw necrosis, considering clinical features of the cases in question. The diagnosis of MRONJ does not require histopathology and this finding does not negate the diagnosis of any cat in the study. While there is no pathognomonic histopathological feature in feline MRONJ patients, histopathology can help rule out other possibilities. Microscopic confirmation of necrotic bone can support the diagnosis of MRONJ in patients with a compatible clinical history.

The relevance of each bacterial isolate from culture and sensitivity in this study is difficult to ascertain. Since the oral cavity is not a sterile environment, many opportunistic oral pathogens are normally present. In the human literature, *Actinomyces* colonies have been suggested to play a role in MRONJ pathogenesis (4). *Actinomyces* spp. was identified in three cases in our study. It is common for microbial cultures from areas of exposed bone to be consistent with isolates of the normal oral microbes (36). However, in the presence of extensive soft tissue involvement and infection, culture and sensitivity enables the clinician to select an appropriate antimicrobial regime for the patient (36). Bacterial colonization of MRONJ tissue samples by diverse bacterial species also emphasizes the plausible relationship to local infection as a triggering event (48). Future studies for feline MRONJ lesions with metagenomic next generation sequencing may allow further understanding of this relationship.

Occurrence of osteomyelitis in histopathology of all patients correlate with radiographic findings and culture results, which is consistent with the human diagnostics findings for MRONJ lesions. While relevant to this disease, our feline study shows that no individual or combination of diagnostic tests is pathognomonic to MRONJ in cats. To differentiate other disease entities causing osteomyelitis and osteonecrosis from MRONJ, it is important that the clinician correlates these diagnostic findings with evidence of chronic clinical bone exposure and medical history indicating the use of a BP.

Timely tooth extraction in patients receiving BP therapy warrants careful consideration. A recent systematic review of the human literature reported triggering factors for MRONJ secondary to BPs, and regardless of administration route, the most significant cause was tooth extractions, followed by spontaneous onset, prosthesis-induced trauma, history of dental surgery, periodontitis (18) and dental implants (6). These factors can adversely affect the immune system and increase the susceptibility to infection (22), perhaps lending credence to the infection hypothesis for MRONJ in people. Several animal studies further support the triggering role of infection in the onset of MRONJ (14, 15, 41). While prosthesis and implants are uncommon in cats, extractions and oral surgery are routinely performed, and periodontal disease is the most diagnosed disease in small animal patients (49). Of importance, in our study and in agreement to people, prior dental extractions were associated with MRONJ lesions in the vast majority of the cat cases.

**Figure 7:**
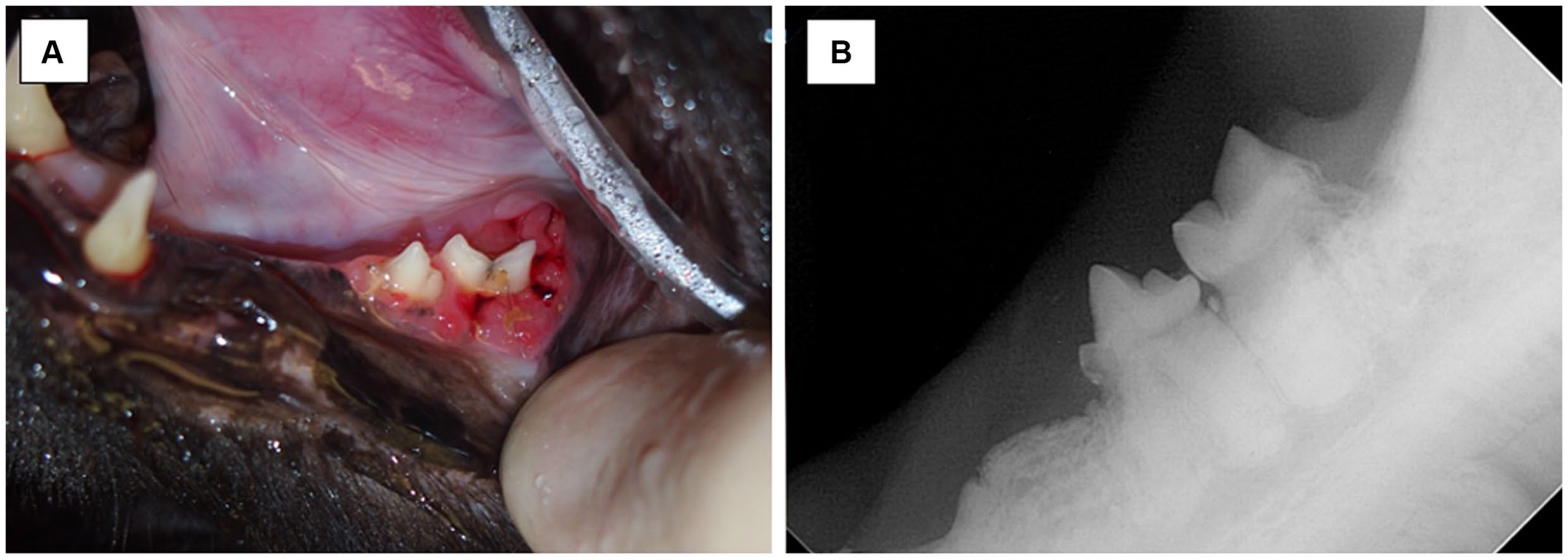
Case 12-Picture **(A)** MRONJ lesion at left mandibular first molar tooth. Chronic trauma from traumatic occlusion from contact of mandibular soft tissue with the cusp of left maxillary fourth premolar tooth and/or tooth resorption suspected to be the inciting cause of lesion. Picture **(B)** Intraoral radiograph of left mandibular fourth premolar tooth and left mandibular first molar tooth. Note the tooth resorption of both left mandibular fourth premolar tooth and left mandibular first molar tooth, and radiographic evidence of irregular bone proliferation at the ventral cortex of the left mandible.

The additional influence of tooth resorption is important to consider in that some cases of MRONJ may arise without any relation to invasive dental surgeries (50). In this study, two cases had teeth with presence of tooth resorption at the site of MRONJ lesion with no prior surgery in that area; one case had tooth resorption and periodontal disease and another had tooth resorption and traumatic malocclusion (Figure 7). Comparatively and of interest, tooth resorption does not appear to be a risk factor for MRONJ in humans. This may mean that the tooth resorption which was noted in these cats was either an incidental finding, secondary to other inflammatory conditions, or the relationship between MRONJ and tooth resorption in cats may differ from humans. Conversely, one study showed that alendronate may delay progression of tooth resorption in cats (30). Further studies need to investigate the role of tooth resorption and MRONJ in cats and may explore an association between MRONJ lesions and subtypes of feline tooth resorption (type 1, type 2 and/or type 3) to highlight more about pathophysiology of the disease.

There is limited evidence that cessation of BPs, or a ‘drug holiday’ prior to oral surgical intervention contributes to preventing MRONJ, and thus no consensus has been reached about this recommendation (1, 5, 43). Our study showed no significant association between stopping bisphosphonate medication prior, during or after surgical treatment and patient outcome. Our study also did not show significant association with outcome between patients who stopped bisphosphonate medication versus those that did not. The half-life of BPs deposited in bone is three years in dogs (51). However, the half-life is unknown in cats. Due to this long half-life, it seems unlikely a short-term discontinuation of medications would prevent MRONJ (4, 52).

**Figure 8:**
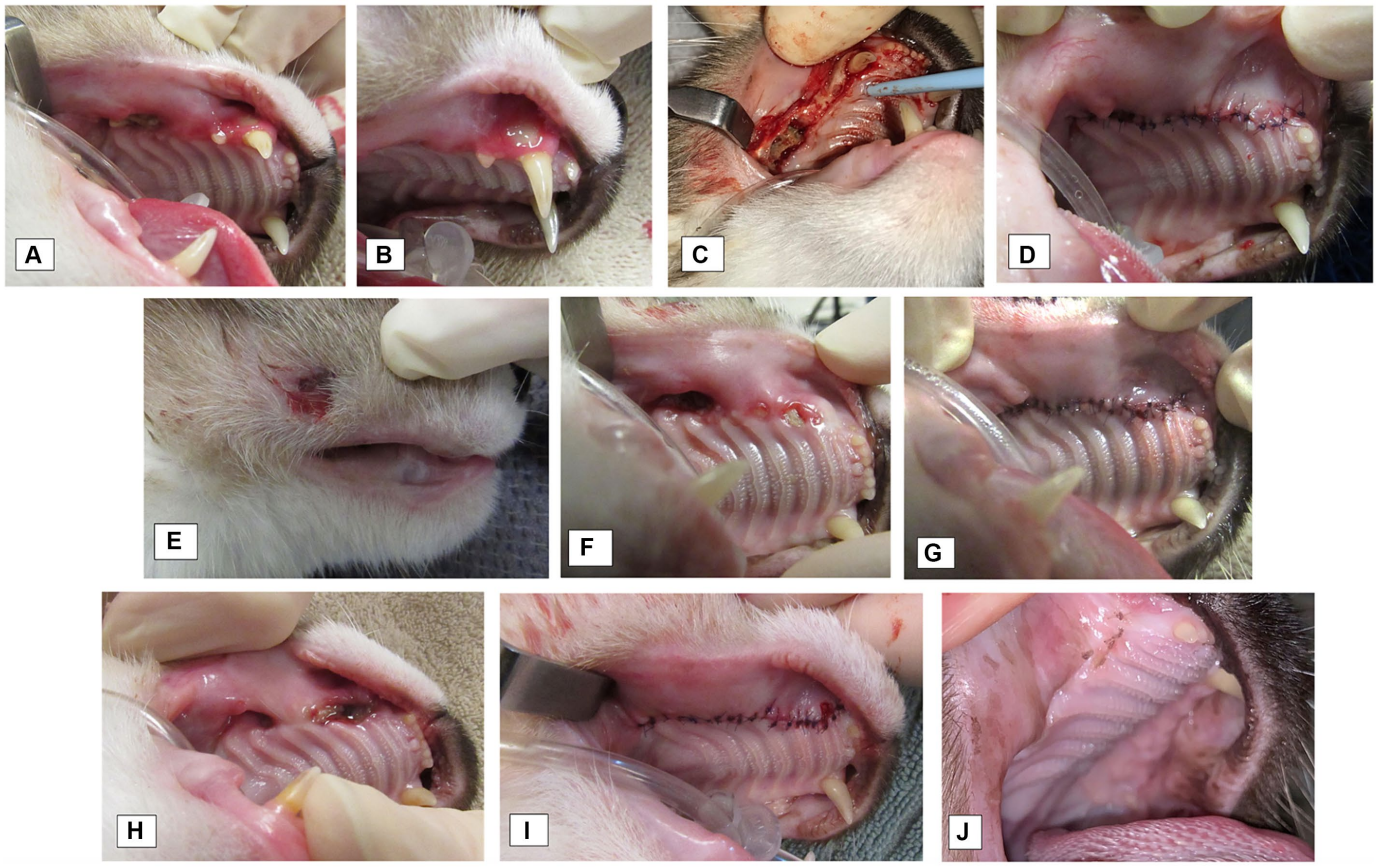
Case 3: Multiple procedures were needed for clinical resolution of MRONJ lesions in the oral cavity. **(A–D)** First procedure to treat MRONJ lesions at right maxilla **(A)** Exposed bone at the alveolus of previously extracted right maxillary second premolar tooth, right maxillary fourth premolar tooth, right maxillary first molar tooth. **(B)** Gingival fenestration with exposed bone at buccal aspect of right maxillary canine tooth. **(C)** Intraoperative view of the necrotic bone at right maxillary fourth premolar tooth extraction alveolus **(D)** Surgical site closure. E-G: Follow up procedure 1 month later. Exposed necrotic bone at the prior surgery site at the right maxillary quadrant. **(E)** Extra-oral draining tract developed **(F)** Intraoral view of non-healing site **(G)** Surgical site closure.H-I: Third procedure 4 months after the second surgery. **(H)** Intraoral view of the non-healing site right maxilla. **(I)** Surgical site closure. **(J)** A small fistula was present 12 months later, and small defect was repaired at missing right maxillary canine tooth. At re-check, site healing well with some remaining suture.

There is no universally accepted treatment protocol for MRONJ in humans and limited published literature in cats. In our study, surgery was necessary for treatment of the lesion in most cases, and approximately a third of the patients who had surgery needed at least one revision procedure performed (Figure 8), highlighting that revision surgeries are common. Surgical debridement was the most common procedure performed, with only one patient undergoing a right mandibulectomy. Goals of treatment in people are three-fold: (1) prevention of extension of MRONJ lesion, (2) maintenance of patient quality of life by relieving symptoms including pain and controlling infection, and (3) patient education and routine follow-up for oral health care with dental experts (4). Treatment is typically divided into conservative or surgical approach. Conservative treatment includes patient education, control of pain and control of secondary infection (1). Surgical methods recommend complete elimination of the lesion and tension-free closure of the surgical wounds (4, 5). The literature highlights a trend in preference for a more aggressive approach in people (4), showing that surgery improved healing rates in all stages with MRONJ, when compared to healing with nonsurgical approaches (43, 53). Our results agree with this statement; though this may be precipitated by the fact that in our veterinary patients, MRONJ diagnosis is typically at later stages of disease when they are already showing clinical signs.

In our study, only one cat had adjuvant treatment; cold laser followed by hyperbaric oxygen therapy. Our study cannot ascertain if the positive outcome in this patient was associated with adjunct treatment. In humans, other novel treatment options which have been described adjuvant to conservative or surgical approaches include hyperbaric oxygen (54), low-level laser treatment (55), platelet-derived growth factor (56), topically applied ozone (57), bone marrow stem cell intralesional transplantation (58), addition of pentoxifylline and tocopherol to standard antibiotic regime (59), fluorescence guided bone resection (60) and teriparatide (N-terminal 34-amino acid recombinant human parathyroid hormone) (61). To understand their safety and efficacy in MRONJ lesions in cats, further studies are warranted.

Based on the human literature, a multimodal approach is recommended to reduce the risk of MRONJ. This includes completing necessary oral surgery and allowing time for bone healing prior to initiating BP treatment. Radiographic evidence of bone formation does not become apparent until 6–8 weeks following tooth extraction (62), so waiting at least 2 months postoperatively prior to starting BP may be a reasonable approach. For patients already on BPs, use of appropriate antimicrobials and antimicrobial mouth rinses before and/or after surgery, appropriate wound closure following tooth extractions, maximizing patient health and maintenance of good oral health prior and during BP treatment are imperative (1, 5, 43). There are no known published recommendations with regards to type of antimicrobial agent or length of treatment in humans or cats with MRONJ. However, long-term use of antimicrobials may be contraindicated due to risk for drug resistance (4). Our study could not derive meaningful conclusions about use of pre or postsurgical antibiotics and patient outcomes. The authors also recommend thorough workup to elucidate whether cause of hypercalcemia is truly idiopathic, and a risk–benefit analysis should be performed when treating cats with BPs for hypercalcemia especially due to significant prevalence of dental disease within the feline population. If BPs are necessary, then client education and communication between all veterinarians managing the patient is essential, along with periodontal treatment every 6–12 months to monitor for oral lesions. The One Health concept recognizes the interrelationship between humans, animals, plants and their universal environment, understanding the transdisciplinary approach to achieve optimal global health outcomes (63). Understanding more about MRONJ in both humans and animals may aid successful treatment and/or prevention of this disease in our feline species moving forward.

Limitations to this study include its retrospective nature, a small number of cases, heterogenous data and lack of a control group; therefore, statistical inferences and meaningful conclusions associated with patient variables and outcome were restricted. Given the nature of acquiring patient history from multiple veterinarians, there is variability to MRONJ diagnostic approach and treatment. Finally, because the diagnosis for idiopathic hypercalcemia in each cat was made by a different veterinarian prior to the patient being evaluated and treated for their MRONJ lesion, there is variation in how this diagnosis was made for each cat, and it is possible there were other underlying causes of the elevated calcium. This study did not address the prevalence of MRONJ secondary to alendronate use in cats. This would be valuable information, particularly for clinicians prescribing the medication. This data would be better addressed with a future prospective cross-sectional study design.

Further studies on MRONJ lesions secondary to BPs in cats are needed, particularly to investigate both the prevalence and incidence of this disease in cats prescribed these medications. Future directions include investigating pertinent imaging findings (ideally utilizing contrast with helical CT) in MRONJ lesions and whether this modality can assist with earlier diagnosis, a microbiologic study focusing on the presence and significance of *Actinomyces* spp. in lesion development and studying the half-life of alendronate in cats.

## Conclusion

The study highlighted multiple pertinent conclusions. Cats treated with alendronate for feline idiopathic hypercalcemia were at risk for developing MRONJ. The most common clinical presentation of MRONJ was a focal lesion in one quadrant of the oral cavity, associated with a prior extraction site. Prior dental extractions were associated with MRONJ lesions in most of the cases. Histopathology, dental imaging and bacterial culture and sensitivity were instrumental in ruling out other disease processes. Treatment of MRONJ lesions include aggressive surgical debridement and appropriate antimicrobial therapy. Prevention of MRONJ is multifactorial. Knowledge that MRONJ can occur with bisphosphonate therapy in cats is critical. Prevention of lesions is equally important, and all dental treatment ideally must occur prior to the institution of these drugs.

To the authors’ knowledge, this is the first report describing a case series of MRONJ secondary to BP treatment in cats and provides valuable information about clinical signs, relevant diagnostic evaluations, differential diagnoses, treatment strategies and preventative options, as well as a current literature review of MRONJ in other species.

## Data Availability

The raw data supporting the conclusions of this article will be made available by the authors, without undue reservation.
