# Supplementary material for: A retrospective case series on bisphosphonate related osteonecrosis of the jaw in 20 cats
Source: Front Vet Sci. 2024 Aug 23;11:1436988. doi: 10.3389/fvets.2024.1436988 (PMC11377274; doi:10.3389/fvets.2024.1436988)
Supplement: Supplementary file 1 [file Table_1.DOCX]

**S1 Table: Culture and Sensitivity Results**

| Patient Number | Testing Performed | Isolate(s) Found | Antibiotic (Abs) Sensitivity to organisms: |
| --- | --- | --- | --- |
| 1 | Aerobic & Anaerobic | Aerobic:  Pasturella spp. (2+)  Burkholderia cepacian (few)  Staphylococcus felis (2+)  No anaerobic organisms isolated. | Enrofloxacin, Marbofloxacin, TMS (for all of the organisms) |
| 3 | Aerobic & Anaerobic | Aerobic:  Pasturella sp. (3+)  Neisseria sp. (1+)  Anaerobic:  Bacteroides sp. (3+) | Aerobic:  Both sensitive to the penicillin’s. Pasturella is sensitive to all of the Abs tested.  Anaerobic: Clindamycin, Metronidazole, Chloramphenicol. |
| 4 | Aerobic & Anaerobic, Fungal | Aerobic:  Escherichia coli (1+)  Actinomyces sp. (1+)  Staphylococcus felis (1+)  No anaerobic organisms isolated.  No fungal growth. | Actinomyces spp. – requires prolonged Abs (penicillin, erythromycin, clindamycin, ampicillin and chloramphenicol)  E. Coli – Sensitive to all Abs.  S. Felis – Sensitive to all Abs. |
| 5 | Aerobic & Anaerobic | Aerobic:  Actinomyces sp. (4+)  No anaerobic organisms isolated. | Actinomyces spp. – requires prolonged Abs (penicillin, erythromycin, clindamycin, ampicillin and chloramphenicol) |
| 6 | Aerobic & Anaerobic | Aerobic:  Bacillus sp. (few)  Actinomyces sp. (few)  No anaerobic organisms isolated. | Bacillus sp. – unknown significance, common environmental organism and in the GI of healthy animals. Therefore susceptibility testing was not performed.  Actinomyces spp. – requires prolonged Abs (penicillin, erythromycin, clindamycin, ampicillin and chloramphenicol) |
| 8 | Aerobic & Anaerobic | Aerobic:  Peptostreptococcus sp. (2+)  Bacteroides sp. (2+)  Anaerobic:  Actinomyces sp. (1+) | Peptostreptococcus sp. – Amoxicillin or Clavamox.  Bacteroides sp. - Clindamycin, metronidazole, chloramphenicol. Penicillin resistant strain.  Actinomyces spp. – requires prolonged Abs (penicillin, erythromycin, clindamycin, ampicillin and chloramphenicol) |
| 14 | Aerobic & Anaerobic | Aerobic:  Escherichia coli (1+)  Corynebacterium sp. (2+)  Micrococcus sp. (2+)  Anaerobic:  Porphyromonas sp. (4+) | Escherichia coli - Sensitive to all Abs.  Corynebacterium sp. – no susceptibility testing was performed.  Micrococcus sp. – environmental organism, usually non-pathogenic.  Porphyromonas sp. – penicillin, ampicillin or Clavamox. |
| 17 | Aerobic & Anaerobic | Anaerobic:  Propionibacterium spp  Prevotella spp. | N/A |
